# Normothermic regional perfusion in controlled donation after circulatory death liver transplantation: an updated systematic review and meta-analysis versus non-NRP and brain-death donors

**DOI:** 10.3389/ti.2026.17154

**Published:** 2026-09-21

**Authors:** José Ramírez‐Esteban, Ezequiel Sarrió, Alberto Ruiz‐Pacheco, Ana Cots, Nekane Romero‐García, Rafael Badenes

**Affiliations:** Department of Anesthesiology and Critical Care, Hospital Clínic Universitari de València, Universitat de València, Valencia, Spain

**Keywords:** normothermic regional perfusion, donation after circulatory death, liver transplantation, ischemic cholangiopathy, graft survival, biliary complications

## Abstract

Normothermic regional perfusion (NRP) may mitigate ischemic injury in controlled donation after circulatory death (cDCD) liver transplantation. We updated the most recent meta-analysis (search closed June 2023) by incorporating subsequent evidence and comparing NRP with non-NRP cDCD and with donation after brain death (DBD). We performed a systematic review of five databases and random-effects meta-analyses; dichotomous outcomes were pooled as risk ratios, the Mantel-Haenszel estimator was preferred for rare events, pooled incidences used a logit-binomial model, risk of bias was assessed with ROBINS-I and certainty with GRADE. Twenty observational studies were included (1776 NRP-cDCD liver recipients). Versus non-NRP cDCD, NRP reduced ischemic cholangiopathy (risk ratio 0.16, 95% confidence interval 0.09–0.28; number needed to treat 9), graft loss (0.41, 0.32–0.54), recipient death (0.46, 0.34–0.62), hepatic artery thrombosis (0.51, 0.30–0.85), other biliary complications (0.49, 0.32–0.73) and primary non-function (0.51, 0.28–0.94); these reductions persisted after excluding grafts managed with *ex situ* machine perfusion. Versus DBD, no outcome differed significantly. Pooled ischemic cholangiopathy incidence was 1.8%, 12.7% and 1.2% with NRP, non-NRP and DBD. NRP markedly reduces ischemic cholangiopathy and improves other outcomes versus non-NRP cDCD, approaching DBD; the evidence remains observational, with moderate-to-serious risk of bias.

**Systematic Review Registration:**

https://www.crd.york.ac.uk/prospero/display_record.php?ID=CRD420261404939, identifier PROSPERO CRD420261404939.

## Graphical Abstract

**Figure:**
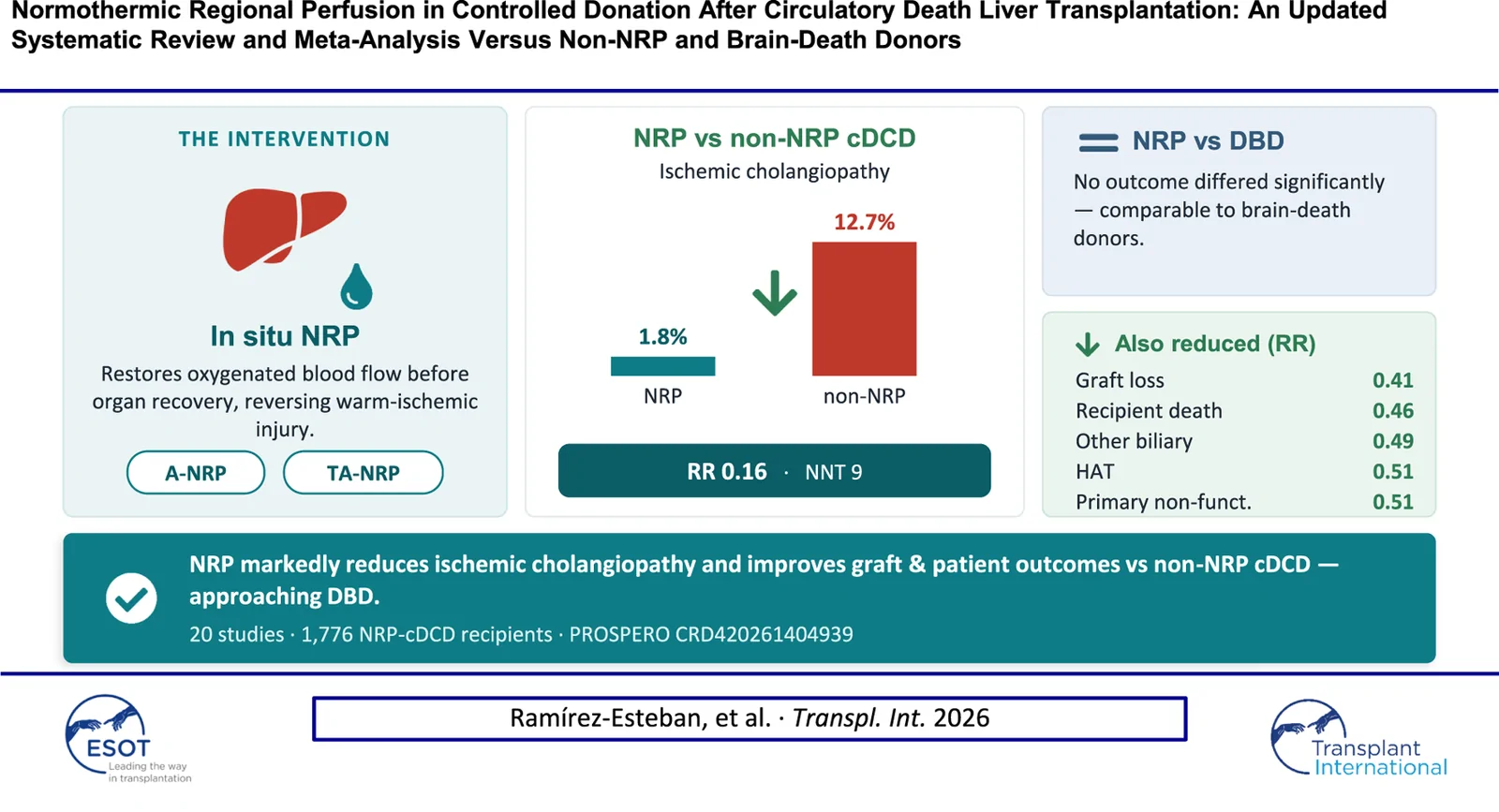

## Introduction

Liver transplantation from controlled donation after circulatory death (cDCD) is now an established means of enlarging the donor pool, but the mandatory interval of warm ischemia that follows withdrawal of life-sustaining treatment and circulatory arrest predisposes to ischemic cholangiopathy (IC), early allograft dysfunction (EAD) and biliary and vascular complications at rates exceeding those of donation after brain death (DBD) [1–3]. IC—non-anastomotic biliary strictures arising in a graft with a patent hepatic artery—is the most feared of these, frequently irreversible, and a leading cause of graft loss and retransplantation in cDCD.

Normothermic regional perfusion (NRP) addresses this injury at its origin. By restoring oxygenated, normothermic blood flow to the abdominal organs *in situ* before procurement, NRP reverses the warm-ischemic insult, replenishes cellular energy substrates and permits functional assessment of the graft before acceptance [3, 4]. The technique may be confined to the abdomen (abdominal NRP, A-NRP) or extended to the thorax for combined cardiothoracic recovery (thoracoabdominal NRP, TA-NRP). It is distinct from *ex situ* machine perfusion, in which the explanted organ is perfused on a device; the two can also be combined sequentially.

NRP programs matured first in Europe—Spain [1, 2, 5–8], the United Kingdom [3, 9], France [10, 11], the Netherlands [12] and Sweden [13]—and have since expanded across the United States, where thoracoabdominal recovery for simultaneous heart procurement has driven uptake [14–18]. Single-center and registry series have consistently reported lower IC and improved graft survival with NRP, and several matched analyses suggested that NRP-cDCD outcomes approach those of DBD [5, 6, 10]. Yet the evidence is fragmented: comparators differ (super-rapid recovery with static cold storage [SCS] or *ex situ* normothermic machine perfusion [NMP]), cohorts overlap (national registries encompassing single-center series; clustered registry data), outcome definitions are inconsistent, and most series are small and single-center.

The most recent systematic review and meta-analysis pooled 11 studies (search closed 9 June 2023) and found that, versus non-NRP cDCD, NRP reduced IC, primary non-function (PNF), graft loss and recipient death, with no difference versus DBD [19]. A subsequent broader systematic review and meta-analysis compared hypothermic, normothermic and regional machine-perfusion strategies and reached concordant conclusions on ischemic cholangiopathy [20], but it evaluated NRP as one of three perfusion techniques and reported only two NRP outcomes, rather than focusing specifically on NRP in cDCD liver transplantation across the full outcome set of the reference review that we update [19]. Since that search the evidence base has nearly doubled, propelled chiefly by large North-American cohorts and registry analyses [15–18]. We therefore performed an updated systematic review and meta-analysis incorporating all subsequent evidence, applying explicit anti-duplication rules for overlapping cohorts and contemporary statistical methods, to deliver the most current and robust estimate of the effect of NRP in cDCD liver transplantation, and to clarify outcomes that remained uncertain in the previous synthesis.

## Materials and methods

### Protocol, eligibility, and search

The review was conducted and reported in accordance with the PRISMA 2020 statement [21] and updated the protocol of the reference meta-analysis [19]; it was prospectively registered (PROSPERO CRD420261404939) and additionally followed the MOOSE recommendations for meta-analyses of observational studies. The PICO framework comprised adult (≥18 years) recipients of a liver graft from Maastricht-III controlled donation after circulatory death (population); graft recovery by *in situ* NRP, whether abdominal (A-NRP) or thoracoabdominal (TA-NRP) (intervention); and two comparators analyzed separately—non-NRP cDCD procurement (super-rapid recovery with static cold storage, with or without *ex situ* HOPE or NMP) and donation after brain death (comparators). Primary outcomes were ischemic cholangiopathy, primary non-function and recipient death; secondary outcomes were graft loss, early allograft dysfunction (Olthoff criteria [22]), hepatic artery thrombosis, other biliary complications (anastomotic strictures and leaks), length of stay and graft utilization.

Eligible studies were randomized trials or comparative cohort studies (prospective or retrospective) reporting at least one primary outcome for NRP-cDCD versus non-NRP cDCD and/or DBD, in adults, published in English or Spanish, with no start-date restriction. We excluded uncontrolled (uDCD) donation without disaggregable cDCD data, studies without a comparator, purely technical descriptions, animal studies, case reports, and conference abstracts with insufficient data, and—consistent with the reference review—grafts from jurisdictions imposing a mandatory no-touch (stand-off) period exceeding 5 min (for example, Italy, 20 min). When cohorts overlapped, the most complete or most recent publication was retained and the decision documented.

Five databases were searched: MEDLINE (via PubMed), Embase, Scopus, the Cochrane Library (CENTRAL) and the Web of Science Core Collection, complemented by hand-searching the reference lists of recent reviews and of the included studies. The strategy combined three concept blocks with the Boolean AND—(i) normothermic regional perfusion, (ii) circulatory-death donation, and (iii) liver and transplantation—deliberately omitting comparator and outcome blocks to preserve sensitivity, with both resolved at screening; the term “ECMO” was admitted only when co-occurring with circulatory-death or donation terms, to retrieve the foundational NRP literature without the noise of cardiorespiratory-support ECMO. Records were managed in Rayyan, de-duplicated, and screened in duplicate at the title/abstract and full-text levels, with disagreements resolved by an arbiter. The full search string for each database and the per-database record counts are provided in the Supplementary Material.

### Data extraction and risk of bias

Two reviewers extracted data independently using a standardized, piloted form that captured study characteristics (design, period, center or registry, country), donor data (age, NRP type and duration, ischemia times), recipient data (age, MELD), and every predefined outcome with its original definition. Extraction was subjected to multichannel verification—triangulation of figures, tables and text; verification of column order against reported percentages (several primary studies list the comparator or DBD arm in the first column); recomputation of every percentage from raw counts; 1:1 reconciliation of sample sizes against the previous meta-analysis; and a second independent extraction. Ischemic cholangiopathy was defined as non-anastomotic biliary strictures in a graft with a patent hepatic artery; “other biliary complications” as anastomotic strictures plus leaks, excluding ischemic cholangiopathy and hepatic artery thrombosis. Risk of bias was assessed independently by two reviewers with ROBINS-I [23], with disagreements resolved by an arbiter and the results displayed as a traffic-light plot; no randomized trial was identified. The certainty of evidence for each outcome was rated with GRADE [24]. As a final quality-control step, an expert clinician performed a final clinical reading of the extracted dataset and of the complete manuscript to confirm the clinical plausibility and internal consistency of all outcomes and conclusions.

### Statistical analysis

The two comparisons were analyzed independently, and abdominal and thoracoabdominal NRP were grouped as “NRP,” as in the reference review. Dichotomous outcomes were pooled as risk ratios (RR) using DerSimonian-Laird random-effects models [25], cross-checked with REML and with Hartung-Knapp; a 0.5 continuity correction was applied only to studies with a zero cell. For rare-event outcomes the Mantel-Haenszel estimator without continuity correction was preferred, with the Peto odds ratio in support, per Cochrane guidance [26]; this mattered for ischemic cholangiopathy versus DBD, where the inverse-variance estimate (RR 1.21) was unstable because of multiple double-zero studies, whereas the Mantel-Haenszel (0.70), Peto (0.70), crude (0.79) and drop-zero (0.91) estimates were concordant and non-significant. Recipient death and graft loss were pooled as RR from counts, too few hazard ratios being available to pool, with individual hazard ratios described narratively. Pooled incidences used a logit-binomial random-effects model, which is more robust than the Freeman-Tukey transformation under high heterogeneity [27]; the latter is reported only as a cross-check. Heterogeneity was summarized with I^2^, τ^2^ and 95% prediction intervals computed with the t-distribution (k−2 degrees of freedom) [28], and continuous outcomes were converted from medians to means by the method of Wan et al. [29]. Prespecified sensitivity (fixed versus random effects, Mantel-Haenszel and Peto, drop-zero, and exclusion of the salvage study and of higher-risk studies) and subgroup (comparator type, region, and use of *ex situ* machine perfusion) analyses were performed, complemented by leave-one-out and Baujat influence diagnostics. To address potential confounding by *ex situ* machine perfusion (normothermic machine perfusion [NMP] or hypothermic oxygenated perfusion [HOPE]), an additional analysis restricted the NRP versus non-NRP comparison to grafts managed without *ex situ* perfusion in either arm; on full-text re-review, one study whose comparator was entirely NMP [11] and one in which dual HOPE was used in both arms [12] were excluded, and a three-arm study contributed through its static-cold-storage arm only, with its NMP arm removed [9]. Publication bias was not formally tested because every analysis included fewer than 10 studies [26]; contour-enhanced funnel plots are provided for completeness (Supplementary Figure S2). Overlapping cohorts were resolved with predefined hierarchical rules applied to each outcome-by-comparison pair: each study competed only within its comparison; national registries served as anchors; the 11 previously included studies were retained; and the new anti-duplication was applied chiefly to the 9 added studies. For GRADE, observational studies began at low certainty and were downgraded for risk of bias, inconsistency (I^2^ > 60%) or imprecision (a confidence interval crossing 1), and upgraded for a large (RR ≤ 0.55) or very large (RR ≤ 0.20) effect, applied cautiously given possible residual confounding. All analyses were reproduced independently in R/metafor and in Python with exact agreement; the complete statistical and anti-duplication decisions, and the per-study data (Supplementary Table S2), are reported in the Supplementary Material.

## Results

### Study selection and characteristics

Of 1,573 records identified, 858 duplicates were removed, 715 were screened, 118 full texts were assessed and 20 studies were included (11 from the previous meta-analysis by Mastrovangelis et al. [19] and 9 published subsequently); 98 reports were excluded with documented reasons (Figure 1) [19]. The included studies comprised 1776 NRP-cDCD liver recipients across the 20 included studies (1,512 in the 16 studies contributing to the meta-analysis) and their non-NRP and DBD comparators across Europe (predominantly Spain) and North America, and ranged from single-center series to national registry cohorts (Table 1). All 20 studies contributed to the qualitative synthesis, whereas 16 contributed to the quantitative synthesis (meta-analysis); the remaining four were retained descriptively but excluded from pooling—two US registry analyses in which NRP could not be isolated or incidence was unreported [14, 15], one Cambridge cohort overlapping an included study [32], and one technique-comparison study [31]. Throughout, non-NRP cDCD comparators comprised the conventional pathway of super-rapid recovery followed by static cold storage (SRR/SCS) unless a study used *ex situ* machine perfusion—normothermic (NMP) or hypothermic oxygenated (HOPE)—which is specified in Table 1.

**FIGURE 1:**
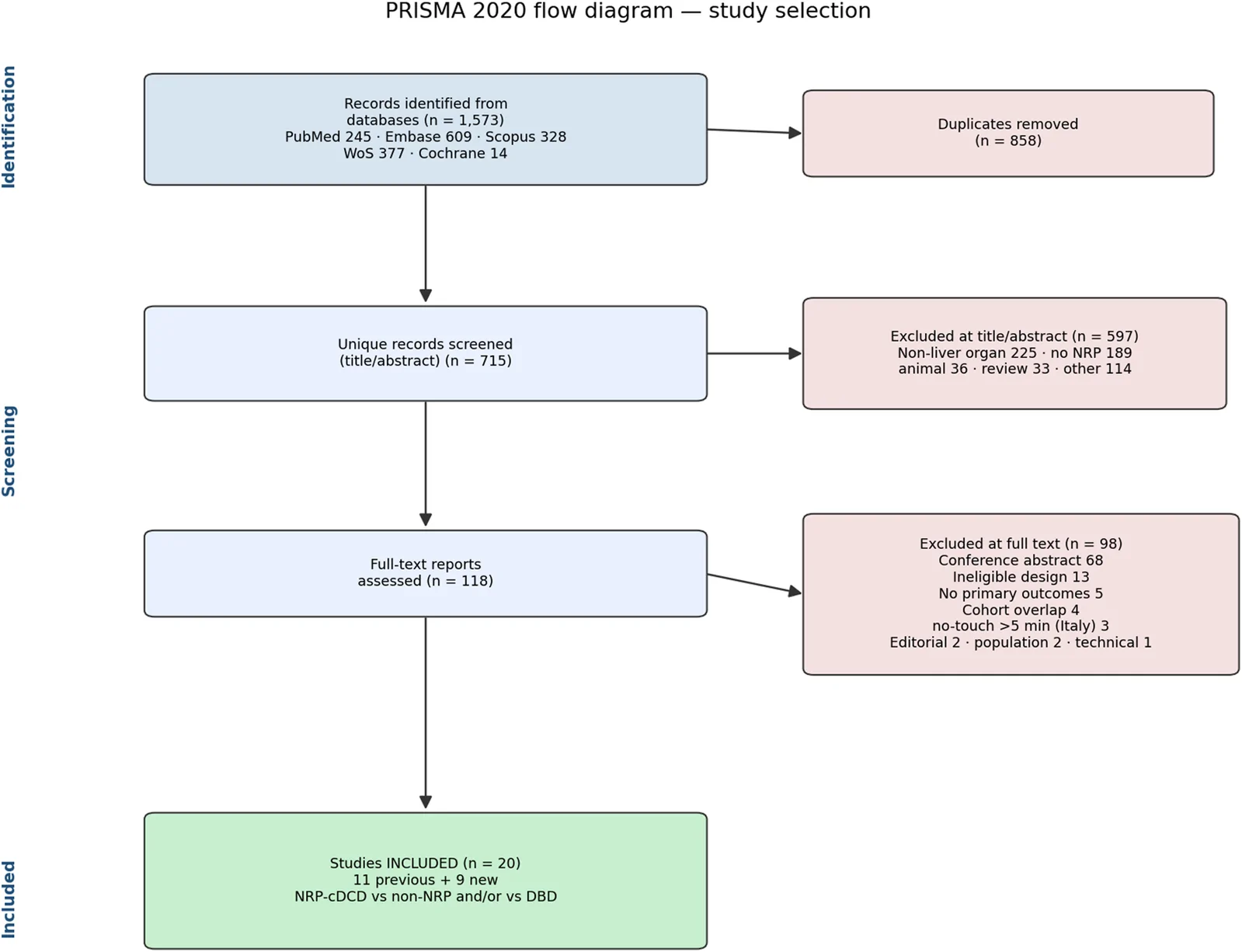
PRISMA 2020 flow diagram of study selection. Of 1,573 records identified across five databases, 858 duplicates were removed, 715 were screened, 118 full texts were assessed, and 20 studies were included (11 from the previous meta-analysis and 9 new). Exclusion reasons are shown at each stage.

**TABLE 1:** Characteristics of the 20 included studies.

Study	Country	Design	Comparison	NRP/comparator (n)	Pooled
Hessheimer 2022 [1]	Spain	National registry	vs. cDCD-SRR/SCS	545/258	Yes
Gaurav 2022 [9]	UK	Single-center	vs. cDCD-SCS and vs. cDCD-NMP (3-arm)	69/97 (SCS) + 67 (NMP)	Yes
Mohkam 2022 [11]	France/UK	Multicenter, PSM	vs. cDCD-NMP	68/34	Yes
Schurink 2022 [12]	Netherlands	Single-center (salvage)	vs. cDCD-SCS and vs. DBD (3-arm, DHOPE)	20/49/81	Yes*§
Rodríguez-Sanjuán 2019 [5]	Spain	Single-center	vs. DBD	11/51	Yes
Miñambres 2020 [2]	Spain	Multicenter	vs. DBD	16/29	Yes
Savier 2020 [10]	France	Multicenter	vs. DBD	50/100	Yes
Ruíz 2021 [6]	Spain	Single-center, matched	vs. DBD	100/200	Yes
Viguera 2021 [7]	Spain	Multicenter	vs. DBD	144/447	Yes
Fernández 2022 [30]	Spain	Single-center, PSM†	vs. DBD	22/51	Yes
Rodríguez 2022 [8]	Spain	Single-center	vs. DBD	39/78	Yes
Brubaker 2024 [16]	USA	Multicenter	vs. cDCD-SRR/SCS	106/136	Yes
Croome 2025 [17]	USA	Multicenter	vs. cDCD-SRR/SCS	62/297	Yes
Campo-Cañaveral 2023 [31]	Spain	National (lung + liver)	vs. DBD	145/1,162	Yes
Bluhme 2024 [13]	Sweden	National pilot, matched	vs. DBD	18/28	Yes
Bababekov 2025 [18]	USA	Single-center	vs. cDCD-SRR/SCS	97/79‡	Yes
Bekki 2023 [14]	USA	Registry (UNOS)	vs. non-NRP cDCD (registry)	24/1,267	No (incidence NR)
Wisel 2023 [15]	USA	Registry (UNOS)	vs. non-NRP cDCD (registry)	133/1,219	No (NRP not isolated)
Secanella 2023 [32]	Spain	Multicenter, matched	TA-NRP vs. A-NRP	6/12	No (technique)
Puttappa 2025 [29]	UK	Single-center	vs. cDCD-SCS or -NMP	101/137	No (overlaps Gaurav)

PSM, propensity-score matching; SCS, static cold storage; NMP, normothermic machine perfusion; SRR, super-rapid recovery; NR, not reported. *Salvage study, included with caution (sensitivity analysis). †NRP, subgroup (n = 22) of a mixed cDCD, cohort. ‡Numbers are recipients transplanted; 86 NRP, and 74 comparator recipients reached 6-month follow-up (the denominator for ischemic cholangiopathy). §Dual HOPE, was used in 25% of NRP, and 40% of comparator grafts; this study was excluded from the *ex situ* perfusion–free sensitivity analysis, in which the three-arm SCS/NRP/NMP, study contributed through its static-cold-storage arm only. Comparator groups: cDCD-SRR/SCS, conventional controlled DCD, pathway (super-rapid recovery followed by static cold storage); cDCD-NMP, *ex situ* normothermic machine perfusion; DHOPE, dual hypothermic oxygenated perfusion; DBD, donation after brain death. In three-arm studies (Gaurav; Schurink) NRP, was compared with each comparator arm separately, with arm sizes shown. In registry studies (Bekki; Wisel) non-NRP cDCD, denotes any non-NRP, procurement, not separable by preservation method.

### Risk of bias

On ROBINS-I, no study was at low risk of bias; 7 were at moderate and 13 at serious risk, driven chiefly by confounding (12/20 serious—inherent to non-randomized designs) and selective reporting (9/20). The two reviewers reached concordant overall judgements (Figure 2). A domain-level summary is provided in Supplementary Figure S1.

**FIGURE 2:**
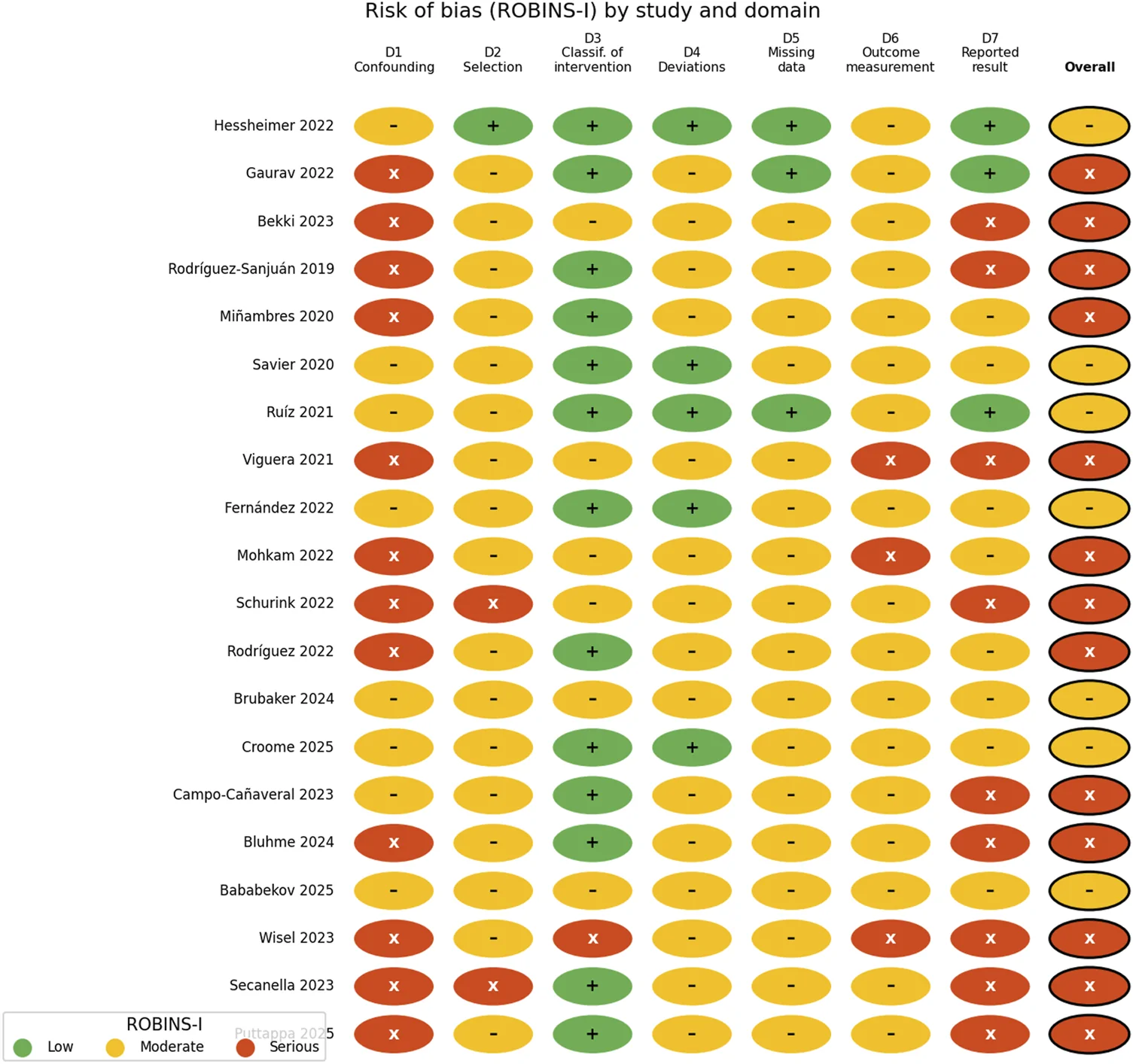
Risk-of-bias assessment (ROBINS-I) by study and domain (D1, confounding; D2, selection of participants; D3, classification of interventions; D4, deviations from intended interventions; D5, missing data; D6, measurement of outcomes; D7, selection of the reported result). Green, low; yellow, moderate; red, serious risk of bias.

### NRP versus Non-NRP cDCD

Between four and seven studies contributed to each pooled comparison—all fewer than ten, which precluded formal small-study (publication-bias) testing [26]. Findings are presented by endpoint (a risk ratio below 1 favours NRP); the pooled estimates are summarized in Figure 3, per-study forest plots appear in Supplementary Figure S4, GRADE summaries in Table 2 and pooled incidences in Table 3.

**FIGURE 3:**
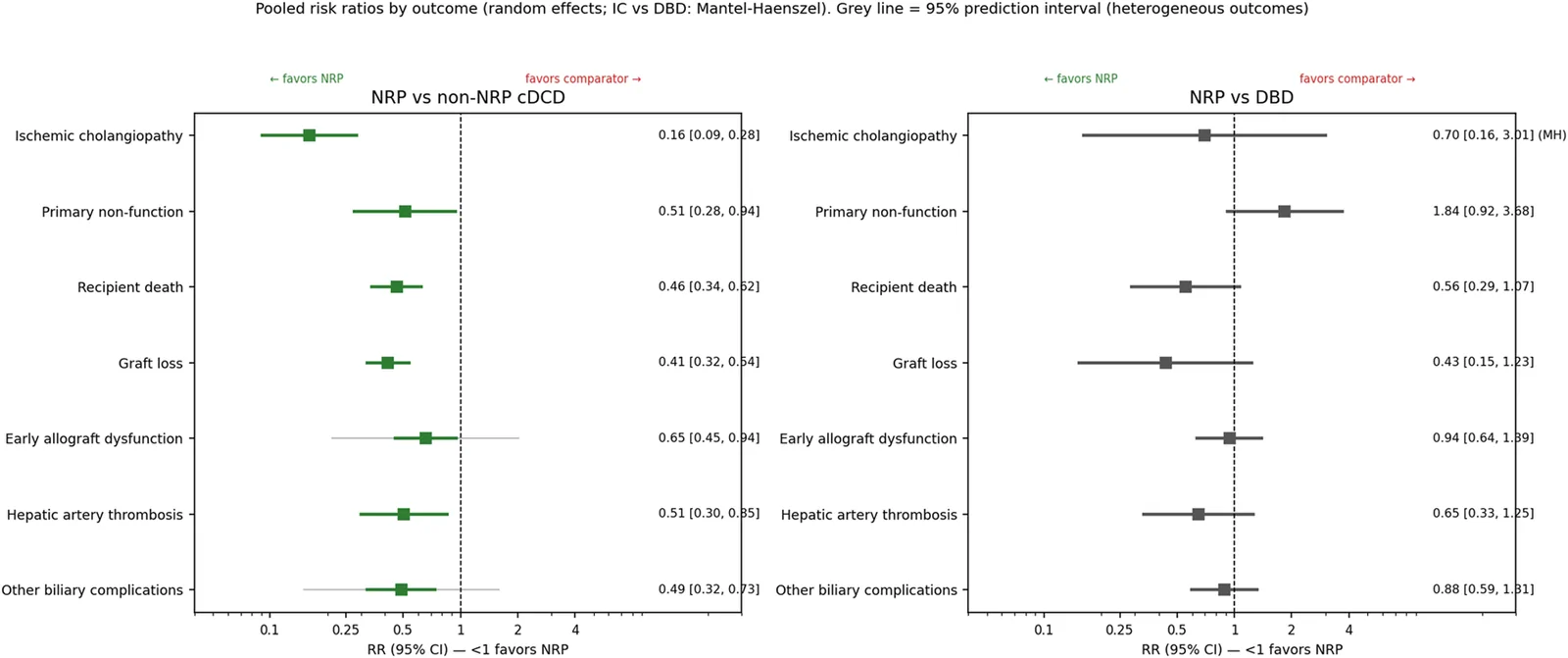
Pooled risk ratios by outcome for both comparisons (NRP vs. non-NRP cDCD, left; NRP vs. DBD, right; random-effects models). A risk ratio below 1 favors NRP. The grey line denotes the 95% prediction interval for heterogeneous outcomes; the ischemic cholangiopathy estimate for the DBD comparison is the Mantel-Haenszel estimate (MH).

**TABLE 2:** Summary of findings (GRADE).

NRP vs. non-NRP cDCD				
Outcome	Studies; n	RR (95% CI)	Absolute/1,000	Certainty
Ischemic cholangiopathy	6; 1878	0.16 (0.09–0.28)	107 fewer	Moderate
Recipient death	4; 1,380	0.46 (0.34–0.62)	38 fewer	Low
Graft loss	5; 1,556	0.41 (0.32–0.54)	56 fewer	Low
Other biliary complications	5; 1,542	0.49 (0.32–0.73)	103 fewer	Low
Hepatic artery thrombosis	6; 1734	0.51 (0.30–0.85)	25 fewer	Low
Primary non-function	6; 1882	0.51 (0.28–0.94)	15 fewer	Low
Early allograft dysfunction	7; 1924	0.65 (0.45–0.94)	Heterogeneous	Very low

NRP vs. DBD				
Outcome	Studies; n	RR (95% CI)	Effect	Certainty
Ischemic cholangiopathy	7; 2055	0.70 (0.16–3.01)‡	No difference	Very low
Primary non-function	8; 2051	1.84 (0.92–3.68)	No difference	Very low
Recipient death	6; 1,266	0.56 (0.29–1.07)	No difference	Very low
Graft loss	4; 613	0.43 (0.15–1.23)	No difference	Very low
Early allograft dysfunction	5; 713	0.94 (0.64–1.39)	No difference	Very low
Hepatic artery thrombosis	8; 2,155	0.65 (0.33–1.25)	No difference	Very low
Other biliary complications	6; 1938	0.88 (0.59–1.31)	No difference	Very low

CI, confidence interval; RR, risk ratio. ‡Mantel-Haenszel estimate (preferred for rare events). Absolute effect = events per 1,000 fewer with NRP, relative to the pooled comparator risk.

**TABLE 3:** Pooled incidences of each outcome by donor and procurement group.

Outcome	NRP, % (95% CI); k	Non-NRP cDCD, % (95% CI); k	DBD, % (95% CI); k
Ischemic cholangiopathy	1.8 (1.2–2.9); 14	12.7 (8.8–18.1); 6	1.2 (0.5–2.8); 7
Recipient death	4.0 (2.1–7.5); 12	7.0 (1.7–24.1); 4	6.4 (3.9–10.5); 6
Graft loss	3.4 (1.4–7.9); 9	9.6 (3.2–25.4); 5	6.0 (3.8–9.4); 4
Other biliary complications	9.5 (7.5–11.9); 11	20.1 (14.6–27.0); 5	10.7 (6.4–17.3); 6
Hepatic artery thrombosis	3.4 (2.4–4.6); 13	5.1 (3.3–7.8); 6	5.1 (3.3–7.8); 8
Primary non-function	3.0 (2.2–4.3); 14	3.0 (1.7–5.2); 6	2.1 (1.5–2.9); 8
Early allograft dysfunction	22.4 (16.0–30.5); 11	35.9 (21.2–53.9); 7	24.7 (19.3–31.0); 5

Values are random-effects logit-binomial pooled incidences (%) with 95% confidence intervals; k, number of contributing studies. These are observed pooled event rates within each group and are not adjusted for between-group differences in case mix. Incidences are pooled over all studies reporting each outcome, whereas relative effects (Table 2) are pooled only over the comparative studies contributing to each comparison; because these study sets differ, the marginal incidences do not reproduce the relative effects—for example, primary non-function shows near-identical marginal incidences (3.0% vs. 3.0%) yet a significant relative effect (RR, 0.51), because within the comparative pool the crude rates are 2.0% versus 3.1%. CI, confidence interval; cDCD, controlled donation after circulatory death; DBD, donation after brain death; NRP, normothermic regional perfusion.

#### Ischemic cholangiopathy

NRP markedly reduced ischemic cholangiopathy (RR 0.16, 95% CI 0.09–0.28; 6 studies; I^2^ = 0%), lowering the pooled incidence from 12.7% without NRP to 1.8% (NNT 9); this non-NRP incidence reproduces the 13.2% of the reference review, an external validation of our dataset. GRADE: moderate certainty.

#### Graft loss

Reduced with NRP (RR 0.41, 0.32–0.54; 5 studies; I^2^ = 0%), from a pooled incidence of 9.6%–3.4% (NNT 18). GRADE: low certainty.

#### Recipient death

Reduced (RR 0.46, 0.34–0.62; 4 studies; I^2^ = 0%), from 7.0% to 4.0% (NNT 27). GRADE: low certainty.

#### Hepatic artery thrombosis

Reduced (RR 0.51, 0.30–0.85; 6 studies; I^2^ = 0%), from 5.1% to 3.4% (NNT 40). GRADE: low certainty.

#### Other biliary complications

Reduced (RR 0.49, 0.32–0.73; 5 studies; I^2^ = 45%, moderate heterogeneity), from 20.1% to 9.5% (NNT 10). GRADE: low certainty.

#### Primary non-function

Reduced (RR 0.51, 0.28–0.94; 6 studies; I^2^ = 0%; NNT 67). The marginal pooled incidence was similar between groups (3.0% vs. 3.0%) because the incidence and relative-effect pools draw on different sets of studies (see Table 3 footnote); within the comparative pool the crude rates were 2.0% versus 3.1%. GRADE: low certainty.

#### Early allograft dysfunction

The only heterogeneous endpoint (RR 0.65, 0.45–0.94; 7 studies; I^2^ = 74%), with pooled incidence falling from 35.9% without NRP to 22.4%. A prespecified subgroup analysis by comparator type (Supplementary Figure S6) showed the reduction was confined to cold-storage or super-rapid-recovery comparators (RR 0.58, 0.39–0.86; 5 studies) and was absent versus *ex situ* normothermic machine perfusion (RR 1.68, 0.82–3.45; 2 studies—Mohkam et al. and the isolated NMP arm of Gaurav et al., 101 NMP recipients) and versus a single hypothermic-oxygenated-perfusion study (RR 0.56, 0.25–1.26). Because the three-arm cohort of Gaurav et al. contributes to two subgroups through its separate comparator arms, the subgroup study counts (2 and 1,5) sum to eight although seven studies enter the overall pool. This subgroup is exploratory and must be read with caution: EAD definitions based on early post-transplant transaminase release (Olthoff, MEAF) are susceptible to biomarker washout during *ex situ* perfusion, which our data cannot separate from a genuine functional effect. GRADE: very low certainty.

#### Length of stay

Intensive-care and hospital stays did not differ significantly (mean differences of about −0.5 to −1.2 days favouring NRP; all confidence intervals crossing zero), with high heterogeneity reflecting differing health systems and reporting.

### NRP versus DBD

Four to eight studies contributed to each endpoint—again fewer than ten, precluding small-study testing—and heterogeneity was low (I^2^ ≤ 4%) for every endpoint except early allograft dysfunction (I^2^ = 34%); no outcome differed significantly and every estimate was of very low certainty by GRADE (Table 2; per-study forest plots in Supplementary Figure S5). Incidences are given as NRP versus DBD.

#### Ischemic cholangiopathy

RR 0.70 (Mantel-Haenszel 0.16–3.01; 7 studies); the inverse-variance estimate (1.21) was unstable because most studies recorded no events. Incidence 1.8% versus 1.2%.

#### Primary non-function

RR 1.84 (0.92–3.68; 8 studies). Incidence 3.0% versus 2.1%.

#### Recipient death

RR 0.56 (0.29–1.07; 6 studies). Incidence 4.0% versus 6.4%.

#### Graft loss

RR 0.43 (0.15–1.23; 4 studies). Incidence 3.4% versus 6.0%.

#### Early allograft dysfunction

RR 0.94 (0.64–1.39; 5 studies; I^2^ = 34%). Incidence 22.4% versus 24.7%.

#### Hepatic artery thrombosis

RR 0.65 (0.33–1.25; 8 studies). Incidence 3.4% versus 5.1%.

#### Other biliary complications

RR 0.88 (0.59–1.31; 6 studies). Incidence 9.5% versus 10.7%.

Overall, NRP recipients had outcomes statistically indistinguishable from DBD, although wide confidence intervals and very low certainty preclude formal equivalence claims.

### Sensitivity analysis excluding ex situ machine perfusion

The NRP-versus-non-NRP comparison was repeated after excluding all grafts managed with *ex situ* perfusion: Mohkam et al. (comparator entirely NMP) and Schurink et al. (dual HOPE in both arms) were removed, and the three-arm cohort of Gaurav et al. contributed through its static-cold-storage arm only. Every effect persisted in the same direction and remained statistically significant, by endpoint: ischemic cholangiopathy RR 0.14 (0.08–0.26; 5 studies; I^2^ = 0%); graft loss 0.41 (0.32–0.53; 4 studies; I^2^ = 0%); recipient death 0.47 (0.35–0.64; 3 studies; I^2^ = 0%); other biliary complications 0.54 (0.37–0.79; 4 studies; I^2^ = 30%); hepatic artery thrombosis 0.48 (0.28–0.83; 4 studies; I^2^ = 0%); primary non-function 0.49 (0.27–0.92; 5 studies; I^2^ = 0%); and early allograft dysfunction 0.58 (0.39–0.86; 5 studies; I^2^ = 78%) (Supplementary Table S3; Supplementary Figure S3). Heterogeneity was therefore unchanged by the exclusion, and the pooled incidences and GRADE certainty reported above apply. The NRP-versus-DBD comparison was inherently free of *ex situ* perfusion. Estimates were also robust to alternative estimators (fixed versus random effects, Mantel-Haenszel, Peto) and to exclusion of the salvage study and of higher-risk studies (Supplementary Table S1). All estimates were reproduced in R/metafor and in Python with exact agreement.

## Discussion

This updated meta-analysis, which expands the evidence base of the most recent NRP-specific synthesis [19], is consistent with and reinforces its central message: in cDCD liver transplantation, NRP substantially reduces ischemic cholangiopathy—the principal liability of cDCD—and is associated with less graft loss, mortality, hepatic artery thrombosis and biliary morbidity than non-NRP procurement. The estimates are remarkably concordant with the previous review (PNF identical at 0.51; recipient death 0.46 vs. a hazard ratio of 0.50), while the larger number of events yields greater precision and, for IC, a somewhat stronger effect (RR 0.16 vs. 0.23) [19]. That two independent datasets, methods and analytic teams converge on the same conclusion is itself reassuring.

Our update offers several contributions. First, it incorporates the emerging North-American experience—registry and multicenter cohorts [15–18]—extending generalizability beyond the predominantly European evidence of the prior review and reproducing the protective signal across health systems and across the A-NRP and TA-NRP techniques. Second, we applied explicit anti-duplication rules to overlapping cohorts that threaten naïve syntheses: Spanish single-center series nested within the national registry [1, 5–8, 30], and a clustered US registry signal [14–18], were prevented from double-counting patients, and a Cambridge cohort overlapping an included study was excluded from pooling [9, 32]. Third, during verification we detected and corrected genuine extraction pitfalls in the primary literature—reversed column order with the comparator listed first [2], evaluable rather than enrolled denominators for EAD [16, 17], a mixed cohort in which only a subgroup received NRP [33], and—prompted by peer review—a salvage cohort in which dual HOPE had been used in both arms [12], which we reclassified accordingly; these errors, left unchecked, would have biased the pooled estimates, and their correction is a methodological contribution in its own right. Reassuringly, restricting the analysis to grafts free of *ex situ* machine perfusion preserved every effect (Supplementary Table S3). Fourth, we used contemporary methods: a logit-binomial model for incidences (avoiding the documented artefacts of the Freeman-Tukey transformation [27]), the Mantel-Haenszel estimator for rare events (avoiding the continuity-correction bias that destabilized the inverse-variance IC-versus-DBD estimate), and t-based prediction intervals [28]. Finally, we report numbers needed to treat for clinical translation and reproduced every estimate in two independent engines.

The heterogeneity of EAD merits emphasis, as it was unresolved (and non-significant) in the previous review [19]. In an exploratory subgroup analysis by comparator type (Supplementary Figure S6), the EAD benefit of NRP was apparent relative to cold storage and super-rapid recovery (RR 0.58, 0.39–0.86) but not relative to *ex situ* normothermic machine perfusion (RR 1.68, 0.82–3.45), the latter based on the two studies that provide isolated NMP comparators (Mohkam et al. and the NMP arm of Gaurav et al.; 101 NMP recipients). Because this rests on only two studies it should be regarded as hypothesis-generating. Importantly, it should not be read as evidence of a biological effect: EAD definitions that incorporate early post-transplant transaminase release (Olthoff, MEAF) are vulnerable to biomarker washout during *ex situ* perfusion, a confounder that our analysis cannot disentangle from any true functional benefit [9, 11]. EAD is therefore comparator-dependent rather than inconsistent—a clinically meaningful distinction that also cautions against pooling perfusion-based and cold-storage comparators indiscriminately. Beyond outcomes, several included studies underline a parallel benefit of NRP on organ utilization, allowing transplantation of grafts that would otherwise be discarded [14, 15]; utilization and quality gains together strengthen the case for NRP at a programmatic level.

Our findings are concordant with, and extend, previous syntheses. An earlier meta-analysis of regional perfusion across DCD solid-organ transplantation [34] and a systematic review of machine-perfusion strategies by donor type [35] reported directionally similar benefits, and a recent broader systematic review and meta-analysis of hypothermic, normothermic and regional perfusion likewise found a marked reduction in ischemic cholangiopathy with NRP versus super-rapid recovery (RR 0.10) [20]. Our contribution is specific rather than duplicative: we focus exclusively on NRP in cDCD liver transplantation with the original three-arm PICO, substantially increase the number of NRP-cDCD recipients relative to the reference NRP-specific review [19], incorporate the emerging North-American registry experience, apply explicit anti-duplication rules, correct extraction errors in the primary literature, use contemporary statistical methods and GRADE, and demonstrate that the findings persist after excluding *ex situ* machine perfusion. Convergence across independent datasets and methods strengthens confidence in the estimates while underscoring their observational nature.

The limitations are intrinsic to the evidence. All studies are observational and at moderate-to-serious risk of bias, dominated by confounding by indication: NRP may be applied to more favorable donor-recipient pairs, which could inflate its apparent benefit. Cohort overlap, although mitigated, cannot be fully excluded without individual-patient data. Events are sparse for several outcomes; fewer than 10 studies contributed to every analysis, precluding formal assessment of publication bias; and death and graft loss were pooled as risk ratios rather than time-to-event hazard ratios. Outcome definitions—particularly of IC and of EAD (Olthoff criteria vs. the MEAF score)—were not uniform [22]. Consequently certainty is moderate at best (for IC) and low to very low otherwise, and the apparent equivalence with DBD reflects absence of evidence of a difference rather than demonstrated equivalence; for IC specifically the DBD comparison was statistically unstable and should be read with caution. Although the sensitivity analysis excluding *ex situ* machine perfusion addresses one important source of confounding, residual confounding by indication cannot be excluded in observational data, and our estimates should be interpreted as associations rather than causal effects.

These caveats notwithstanding, the consistency, magnitude and graded nature of the IC reduction (a number needed to treat of about 9, with incidence falling from 12.7% to DBD-like levels of <2%) are clinically compelling and biologically coherent. Future priorities include randomized and registry-embedded pragmatic trials, a harmonized definition of ischemic cholangiopathy, individual-patient-data meta-analysis to definitively resolve cohort overlap and to compare A-NRP with TA-NRP, and longer follow-up to confirm both the durability of the benefit and the approximation to DBD outcomes.

## Conclusion

In controlled DCD liver transplantation, NRP is associated with a marked reduction in ischemic cholangiopathy (moderate certainty) and with improvements in mortality, graft loss and vascular and biliary complications (low certainty), with results that approach those of DBD (very low certainty). This updated synthesis—building on prior work with a larger and more geographically diverse evidence base—reinforces the role of NRP in expanding the cDCD donor pool, while underscoring the observational nature of the evidence and the need for higher-certainty studies.

## Data Availability

The data analyzed in this study is subject to the following licenses/restrictions: Available from the corresponding author on reasonable request. Requests to access these datasets should be directed to Alberto Ruiz Pacheco, albertoruizp96@gmail.com.
