# Supplementary material for: Normothermic regional perfusion in controlled donation after circulatory death liver transplantation: an updated systematic review and meta-analysis versus non-NRP and brain-death donors
Source: Transpl Int. 2026 Sep 21;39:17154. doi: 10.3389/ti.2026.17154 (PMC13634986; doi:10.3389/ti.2026.17154)
Supplement: Supplementary file 1 [file Supplementaryfile1.docx]

**Supplementary Material**

PROSPERO CRD420261404939. The numbering of supplementary items is referenced from the main text.

# Contents

**Supplementary Methods 1.** Full electronic search strategy, by database

**Supplementary Methods 2.** Cohort-overlap and anti-duplication rules, with per-study decisions

**Supplementary Methods 3.** Statistical methods (full detail)

**Supplementary Methods 4.** Data-verification and reproducibility audit

**Supplementary Methods 5.** Primary-data errors detected and corrected during verification

**Supplementary Figure S1.** ROBINS-I risk-of-bias summary (domain-level)

**Supplementary Figure S2.** Contour-enhanced funnel plots

**Supplementary Figure S3.** Forest plot of the NRP versus non-NRP cDCD comparison restricted to grafts free of ex situ machine perfusion

**Supplementary Figure S4**. Per-study forest plots, NRP versus non-NRP cDCD.

**Supplementary Figure S5**. Per-study forest plots, NRP versus DBD.

**Supplementary Figure S6**. Early allograft dysfunction by comparator type (NRP versus non-NRP cDCD).

**Supplementary Table S1.** Sensitivity analyses, heterogeneity and influence diagnostics

**Supplementary Table S2.** Per-study raw data (events/totals) and reported hazard ratios

**Supplementary Table S3.** Sensitivity analysis excluding ex situ machine perfusion (NMP/HOPE)

**Supplementary Checklist 1.** PRISMA 2020 checklist

**Supplementary Checklist 2.** MOOSE checklist

**Capsule Sentence Summary**

**Graphical Abstract (journal template)**

# Supplementary Methods 1. Full electronic search strategy

Five databases were searched on 25 May 2026 with no start-date restriction, replicating and extending the strategy of the reference meta-analysis. The strategy combined three concept blocks with AND: (1) normothermic regional perfusion, (2) circulatory-death donation, and (3) liver and transplantation. No comparator or outcome block was used in the syntax, to preserve sensitivity; both were resolved at screening. “ECMO” was admitted only in co-occurrence with circulatory-death or donation terms, to retrieve the foundational NRP literature without the noise of cardiorespiratory-support ECMO. An English-language limit was applied, consistent with the reference review. Records were imported to Rayyan, de-duplicated, and screened in duplicate with arbitration.

**Records retrieved per database (final search, 25 May 2026)**

| **Database** | **Search date** | **Records** |
| --- | --- | --- |
| MEDLINE (via PubMed) | 25 May 2026 | 245 |
| Embase | 25 May 2026 | 609 |
| Scopus | 25 May 2026 | 328 |
| Web of Science Core Collection | 25 May 2026 | 377 |
| Cochrane CENTRAL | 25 May 2026 | 14 |
| Hand-searching of references | 25 May 2026 | 0 |
| Total (before de-duplication) |  | 1,573 |

*Counts correspond to the PRISMA 2020 flow diagram (Figure 1). 858 duplicates were removed, leaving 715 unique records screened by title/abstract; 118 full texts were assessed and 20 studies were included.*

## A. MEDLINE (PubMed) — MeSH and free text

#1 NRP block:

("normothermic regional perfusion"[tiab] OR "regional perfusion"[tiab] OR NRP[tiab] OR "A-NRP"[tiab] OR "TA-NRP"[tiab] OR "in situ perfusion"[tiab] OR ((ECMO[tiab] OR "extracorporeal membrane oxygenation"[tiab]) AND ("circulatory death"[tiab] OR "cardiac death"[tiab] OR DCD[tiab] OR cDCD[tiab] OR "non-heart-beating"[tiab] OR asystol*[tiab] OR Maastricht[tiab])))

#2 Circulatory-death block:

("Tissue and Organ Procurement"[Mesh] OR "donation after circulatory death"[tiab] OR "donation after cardiac death"[tiab] OR "circulatory death"[tiab] OR "cardiac death"[tiab] OR DCD[tiab] OR cDCD[tiab] OR "non-heart-beating"[tiab] OR "non heart beating"[tiab] OR asystol*[tiab] OR Maastricht[tiab])

#3 Liver + transplant block:

(("Liver Transplantation"[Mesh] OR "Liver"[Mesh] OR liver[tiab] OR hepatic[tiab]) AND ("Transplantation"[Mesh] OR transplant*[tiab] OR graft*[tiab] OR allograft*[tiab]))

#4 Combination and filter: #1 AND #2 AND #3 — Filters: English[lang]

## B. Embase (Emtree + free text)

#1 NRP block:

('normothermic regional perfusion':ab,ti OR 'regional perfusion':ab,ti OR NRP:ab,ti OR 'abdominal normothermic':ab,ti OR 'thoracoabdominal normothermic':ab,ti OR 'A-NRP':ab,ti OR 'TA-NRP':ab,ti OR 'in situ perfusion':ab,ti OR (('extracorporeal oxygenation'/exp OR ECMO:ab,ti OR 'extracorporeal membrane oxygenation':ab,ti) AND ('circulatory death':ab,ti OR 'cardiac death':ab,ti OR DCD:ab,ti OR cDCD:ab,ti OR asystol*:ab,ti OR Maastricht:ab,ti)))

#2 Circulatory-death block:

('organ procurement'/exp OR 'donation after circulatory death':ab,ti OR 'donation after cardiac death':ab,ti OR 'circulatory death':ab,ti OR 'cardiac death':ab,ti OR DCD:ab,ti OR cDCD:ab,ti OR 'non heart beating':ab,ti OR asystol*:ab,ti OR Maastricht:ab,ti)

#3 Liver + transplant block:

(('liver transplantation'/exp OR 'liver'/exp OR liver:ab,ti OR hepatic:ab,ti) AND ('transplantation'/exp OR transplant*:ab,ti OR graft*:ab,ti OR allograft*:ab,ti))

#4 Combination and filter: #1 AND #2 AND #3 AND [english]/lim

## C. Scopus (TITLE-ABS-KEY)

( TITLE-ABS-KEY ( "normothermic regional perfusion" OR "regional perfusion" OR NRP OR "A-NRP" OR "TA-NRP" OR "in situ perfusion" OR ( ( ECMO OR "extracorporeal membrane oxygenation" ) AND ( "circulatory death" OR "cardiac death" OR DCD OR cDCD OR asystol* OR Maastricht ) ) ) ) AND ( TITLE-ABS-KEY ( "donation after circulatory death" OR "donation after cardiac death" OR "circulatory death" OR "cardiac death" OR DCD OR cDCD OR "non-heart-beating" OR asystol* OR Maastricht ) ) AND ( TITLE-ABS-KEY ( ( liver OR hepatic ) AND ( transplant* OR graft* OR allograft* ) ) ) AND ( LIMIT-TO ( LANGUAGE , "English" ) )

## D. Cochrane CENTRAL (Ovid/Wiley syntax)

```
#1 ("normothermic regional perfusion" OR "regional perfusion" OR NRP OR "A-NRP" OR "TA-NRP" OR "in situ perfusion"):ti,ab,kw
#2 ("donation after circulatory death" OR "circulatory death" OR "cardiac death" OR DCD OR cDCD OR "non-heart-beating" OR asystol* OR Maastricht):ti,ab,kw
#3 ((liver OR hepatic) AND (transplant* OR graft* OR allograft*)):ti,ab,kw
#4 Combination: #1 AND #2 AND #3
```

***Note.*** *In CENTRAL, given the low prevalence of trials, ECMO was left unrestricted to maximize sensitivity; the expected volume is low.*

## E. Web of Science Core Collection (TS syntax)

TS=("normothermic regional perfusion" OR "regional perfusion" OR NRP OR "A-NRP" OR "TA-NRP" OR "in situ perfusion" OR ((ECMO OR "extracorporeal membrane oxygenation") AND ("circulatory death" OR "cardiac death" OR DCD OR cDCD OR asystol* OR Maastricht)))

AND TS=("donation after circulatory death" OR "circulatory death" OR "cardiac death" OR DCD OR cDCD OR "non-heart-beating" OR asystol* OR Maastricht)

AND TS=((liver OR hepatic) AND (transplant* OR graft* OR allograft*)) — Refine: Languages = English

# Supplementary Methods 2. Cohort-overlap and anti-duplication rules

Two comparisons were analyzed independently: NRP versus non-NRP cDCD (super-rapid recovery / static cold storage, with or without ex situ HOPE/NMP) and NRP versus DBD. A patient contributing to one pool was never counted again within the same pool. Abdominal and thoracoabdominal NRP were grouped as “NRP,” as in the reference review.

**Hierarchical priority rule.** The unit of decision was the outcome × comparison pair, not the whole study: a study could be retained for one outcome/comparison and dropped for another. (1) Each study competed only within its comparison. (2) With overlapping patients in the same comparison, a single source was kept, prioritizing the most complete primary-outcome data (IC/PNF/death), the largest size, and the most recent publication; national registries acted as anchors within their comparison. (3) The 11 studies of the previous review were retained as there. (4) New anti-duplication was applied chiefly to the 9 added studies.

## Per-study decisions

| **Study (studies)** | **Decision** | **Rationale** |
| --- | --- | --- |
| Hessheimer 2022 | Include — non-NRP anchor | Spanish national registry; the Spanish single-center series sit in the NRP-vs-DBD pool (a different comparison) → no double counting. |
| Gaurav 2022 | Include — Cambridge anchor | Reports the IC primary outcome; takes priority over Puttappa (same program). |
| Bekki 2023 | Exclude from pools | UNOS; IC/PNF only as a cause of loss (incidence not reported); n=24. Descriptive only. |
| Rodríguez-Sanjuán 2019; Miñambres 2020; Savier 2020; Ruíz 2021; Viguera 2021; Fernández 2022; Mohkam 2022; Rodríguez 2022 | Include (as in previous review) | Studies from the previous review; Spanish overlap accepted and documented as a limitation. |
| Schurink 2022 | Include with caution | Salvage study (A-NRP on discarded grafts), mixed comparator; enters both comparisons (vs SCS 49 and vs DBD 81), flagged for sensitivity. |
| Brubaker 2024; Croome 2025; Bababekov 2025 | Include in pools | Distinct US institutions (consortium / Mayo+Tampa / Colorado); no demonstrable patient overlap; clean outcomes. |
| Campo-Cañaveral 2023 | Include (NRP vs DBD) | Spanish national cohort (lung+liver); registry overlap flagged as a limitation. |
| Bluhme 2024 | Include | Independent Swedish national cohort. |
| Wisel 2023 | Exclude from pools | TA-DCD mixes NRP+DPP (NRP effect not isolable) plus high UNOS overlap. Descriptive only. |
| Secanella 2023 | Exclude from pools | Technique comparison TA-NRP vs A-NRP (both arms NRP). |
| Puttappa 2025 | Exclude from pools | Same Cambridge NRP program as Gaurav (overlapping patients). Narrative/sensitivity. |

# Supplementary Methods 3. Statistical methods (full detail)

**Effect measures.** Dichotomous outcomes (IC, PNF, EAD, HAT, other biliary) were pooled as risk ratios (RR). Recipient death and graft loss were pooled as RR from counts because too few hazard ratios were reported to pool; individual hazard ratios are described narratively. The previous review used hazard ratios for these two outcomes; our RR estimates are concordant in magnitude and direction.

**Model.** DerSimonian-Laird random effects (comparable to the previous review), cross-checked with REML and with Hartung-Knapp. Heterogeneity was summarized with I², τ² and a prediction interval computed with the t-distribution (k−2 degrees of freedom; Riley 2011 / IntHout 2014), which is more conservative than the normal approximation that metafor uses by default with DL.

**Rare events / zero cells.** A 0.5 continuity correction was applied only to studies with a zero cell (to=“only0”). For rare-event outcomes the preferred estimator was Mantel-Haenszel without continuity correction (with Peto OR in support), per the Cochrane Handbook, being robust to zero cells. IC vs DBD: three double-zero studies; inverse-variance DL gives RR 1.21 but is unstable (the 0.5 correction reverses the direction), whereas Mantel-Haenszel gives RR 0.70 (0.16–3.01), Peto 0.70, crude rate 0.79 and drop-zero 0.91 — all concordant and non-significant. The Mantel-Haenszel estimate is reported as the preferred point estimate for this outcome.

**Pooled incidences.** Primary method: random-effects logit-binomial model. The Freeman-Tukey double-arcsine transformation is reported only as a cross-check with the previous review, because under high heterogeneity it produced misleading incidences (e.g., IC-NRP yielded 0.3% when the true value is ~1.8%). With the logit model, non-NRP IC incidence was 12.7%, reproducing the 13.2% of the previous review.

**Continuous outcomes.** ICU/hospital stay: median[IQR] or mean[range] converted to mean and standard deviation by the method of Wan (2014); the combined SCS+NMP comparator of one study was merged with the group-pooling formula; random-effects mean-difference synthesis.

**Publication bias.** Not formally tested: every analysis included <10 studies (maximum k=8), below the threshold at which Cochrane guidance considers Egger’s test and trim-and-fill unreliable. Contour-enhanced funnel plots are provided as exploratory visual support (Figure S2).

**Certainty (GRADE).** Observational studies start at “low” certainty; downgraded for risk of bias (confounding), inconsistency (I²>60%) and imprecision (CI crossing 1); upgraded for large (RR≤0.55) or very large (RR≤0.20) effects, applied cautiously given possible residual confounding.

**Reproducibility.** All analyses were reproduced independently in R/metafor and Python, with exact agreement, and re-derived a third time from the source sheet.

# Supplementary Methods 4. Data-verification and reproducibility audit

## Extraction verification

- Six-channel verification per study: triangulation of image + table + text (tables read as rendered images, not only the text layer); column-order check against reported percentages (several studies list the comparator/DBD in the first column); cross-percentage recomputation of every percentage from events/N to match the reported figure; automated table extraction; 1:1 reconciliation against the previous review; and a second independent extraction.
- 1:1 reconciliation of sample sizes against Tables 2 and 3 of the reference review: 12/12 comparisons of the 11 overlapping studies matched exactly.
- Blind re-extraction by independent second reviewers of a sample of new studies: one cohort matched 4/4 outcomes; in another, the second reviewer made an arithmetic slip on EAD that the cross-percentage check refuted, confirming the original value. Documented lesson: no single channel is infallible; triangulation of ≥2 channels yields the correct answer.

## Analysis verification

- Triple computation engine: every pooled estimate was computed in R/metafor, in independent Python, and by a third re-derivation from the source sheet — exact agreement across all 14 pools (maximum difference 0.000).
- Cross-consistency between spreadsheet tabs (N per study across tabs, events ≤ N, no text in numeric columns): zero discrepancies.
- Direction sanity (sign of the pooled RR matches the crude rates) and influence/outlier diagnostics (leave-one-out + Baujat) across all 14 pools: the single outlier (one study’s EAD) did not change the direction of any result.
- External validation: non-NRP IC incidence (12.7%) reproduces the 13.2% of the previous review; all estimates reproduce the pattern of the previous review.

Ex situ machine-perfusion re-review: the preservation method of every included study was re-verified against the full text; only three pooled studies involved ex situ perfusion (9, 11, 12), handled in the ex situ perfusion–free sensitivity analysis (Table S3, Figure S3).

## Report verification

- Cross-consistency between deliverables: table values (RR, k, n, absolute effects, certainty) agree across the spreadsheet, the results report and the manuscript.
- Final numerical audit of the manuscript and report (extraction of every figure and reconciliation with sources) corrected two manuscript figures written without calculation (total NRP grafts, corrected to 1,776; and the IC absolute effect, corrected from 127 to 107 fewer per 1,000).
- ROBINS-I and GRADE were assessed independently by two reviewers with no discrepancy in overall judgements.

# Supplementary Methods 5. Primary-data errors detected and corrected

Multichannel verification detected and corrected the following genuine extraction pitfalls in the primary literature; uncorrected, these would have biased the pooled estimates.

| **Study** | **Correction** |
| --- | --- |
| Miñambres 2020 | EAD corrected for column order (DBD-first table) → cDCD 3/16, DBD 5/29. |
| Savier 2020 | IC = disseminated biliary stricture 1/50 vs 1/100; HAT = thrombosis 0/50 vs 5/100 (not “arterial complications”). |
| Fernández-de la Varga 2022 | NRP subgroup n=22 of a mixed cohort (29 SRR + 22 NRP). EAD and death → not reported at subgroup level (cohort-level or Kaplan-Meier only). IC/PNF/HAT/biliary attributed to the subgroup per the text. |
| Brubaker 2024 | EAD: the table’s “77/123” were evaluable denominators; true events 28/77 vs 69/123 (confirmed in the supplement). |
| Bluhme 2024 | cDCD-NRP n=18 (not 28); PNF was in the DBD column; non-anastomotic strictures 0/18 vs 2/28. |
| Croome 2025 | SCS n=297 (not 287); evaluable EAD denominator 187/290 (=64.5%). |
| Schurink 2022 | Added the missing vs-DBD comparison (20 vs 81) to align with the previous review. On full-text re-review (prompted by peer review), DHOPE was used in 25% of NRP and 40% of comparator grafts; reclassified as ex situ perfusion–exposed and excluded from the ex situ perfusion–free sensitivity analysis (Table S3). |

**Supplementary Figure S1. Domain-level ROBINS-I risk-of-bias summary.**

*Proportion of included studies at low, moderate and serious risk of bias for each ROBINS-I domain. No study was at low overall risk; confounding and selective reporting were the dominant domains. The study-level traffic-light plot is Figure 2 of the main text.*

**Supplementary Figure S2. Contour-enhanced funnel plots.**

*Exploratory contour-enhanced funnel plots for the principal pooled outcomes. Formal small-study tests (Egger, trim-and-fill) were not performed because every analysis included fewer than 10 studies; the plots are provided for visual inspection only.*

**Supplementary Figure S3. Forest plot of the NRP versus non-NRP cDCD comparison restricted to grafts free of ex situ machine perfusion.**

Pooled risk ratios (random-effects) after excluding all grafts managed with ex situ perfusion: the study whose comparator was entirely NMP (11) and the salvage study using dual HOPE in both arms (12) were excluded, and the three-arm SCS/NRP/NMP cohort (9) contributed through its static-cold-storage arm only. A risk ratio below 1 favours NRP.

**Supplementary Figure S4. Per-study forest plots, NRP versus non-NRP cDCD.**

Random-effects risk ratios (DerSimonian-Laird) for each outcome; squares, study estimates (area ∝ weight); red diamonds, pooled estimates with 95% CI; k and I² per outcome.

**Supplementary Figure S5. Per-study forest plots, NRP versus DBD.**

Random-effects risk ratios for each outcome in the NRP-versus-DBD comparison; conventions as in Figure S4.

**Supplementary Figure S6. Early allograft dysfunction by comparator type (NRP versus non-NRP cDCD).**

Subgroup forest plot: NRP versus cold storage/super-rapid recovery, versus ex situ normothermic machine perfusion (NMP; Mohkam and the isolated NMP arm of Gaurav, 101 comparator recipients; pooled RR 1.68, 95% CI 0.82–3.45), and versus dual hypothermic oxygenated perfusion (HOPE; single study). Subtotals are random-effects estimates and are exploratory.

**Supplementary Table S1. Sensitivity analyses, heterogeneity and influence diagnostics**

*Pooled risk ratios under alternative estimators and diagnostics for each outcome and comparison. DL, DerSimonian-Laird (primary); REML-HK, REML with Hartung-Knapp; MH, Mantel-Haenszel; Peto, Peto odds ratio; PI, 95% prediction interval; LOO, leave-one-out range of the pooled point estimate. For rare-event outcomes the MH estimate is preferred (see Supplementary Methods 3).*

| **Comparison** | **Outcome** | **k** | **RR DL (95% CI)** | **REML-HK (95% CI)** | **MH** | **Peto OR** | **I² %** | **PI** | **LOO range** |
| --- | --- | --- | --- | --- | --- | --- | --- | --- | --- |
| vs non-NRP cDCD | Ischemic cholangiopathy | 6 | 0.161 (0.091–0.283) | 0.161 (0.085–0.303) | 0.126 | 0.197 | 0 | 0.091–0.283 | 0.126–0.199 |
| vs non-NRP cDCD | Primary non-function | 6 | 0.512 (0.277–0.943) | 0.512 (0.285–0.918) | 0.426 | 0.435 | 0 | 0.277–0.943 | 0.468–0.538 |
| vs non-NRP cDCD | Recipient death | 4 | 0.463 (0.343–0.624) | 0.463 (0.346–0.62) | 0.459 | 0.373 | 0 | 0.343–0.624 | 0.418–0.47 |
| vs non-NRP cDCD | Graft loss | 5 | 0.415 (0.322–0.535) | 0.415 (0.333–0.517) | 0.41 | 0.318 | 0 | 0.322–0.535 | 0.408–0.424 |
| vs non-NRP cDCD | Early allograft dysfunction | 7 | 0.654 (0.453–0.945) | 0.657 (0.349–1.239) | 0.607 | 0.48 | 73.9 | 0.277–1.547 | 0.601–0.755 |
| vs non-NRP cDCD | Hepatic artery thrombosis | 6 | 0.505 (0.3–0.85) | 0.505 (0.317–0.805) | 0.446 | 0.443 | 0 | 0.3–0.85 | 0.388–0.542 |
| vs non-NRP cDCD | Other biliary complications | 5 | 0.488 (0.324–0.733) | 0.486 (0.266–0.888) | 0.501 | 0.436 | 45.2 | 0.236–1.007 | 0.433–0.548 |
| vs DBD | Ischemic cholangiopathy | 7 | 1.206 (0.386–3.766) | 1.206 (0.57–2.554) | 0.699 | 0.705 | 0 | 0.386–3.766 | 1.068–1.525 |
| vs DBD | Primary non-function | 8 | 1.837 (0.917–3.678) | 1.837 (0.878–3.844) | 1.598 | 1.771 | 0 | 0.917–3.678 | 1.589–2.052 |
| vs DBD | Recipient death | 6 | 0.556 (0.289–1.069) | 0.545 (0.234–1.271) | 0.501 | 0.536 | 3.8 | 0.268–1.154 | 0.485–0.723 |
| vs DBD | Graft loss | 4 | 0.435 (0.153–1.232) | 0.435 (0.122–1.551) | 0.338 | 0.413 | 0 | 0.153–1.232 | 0.348–0.535 |
| vs DBD | Early allograft dysfunction | 5 | 0.943 (0.638–1.392) | 0.943 (0.55–1.615) | 0.914 | 0.892 | 34.5 | 0.498–1.786 | 0.791–1.082 |
| vs DBD | Hepatic artery thrombosis | 8 | 0.648 (0.334–1.255) | 0.648 (0.4–1.048) | 0.501 | 0.549 | 0 | 0.334–1.255 | 0.528–0.703 |
| vs DBD | Other biliary complications | 6 | 0.884 (0.594–1.314) | 0.884 (0.752–1.038) | 0.872 | 0.862 | 0 | 0.594–1.314 | 0.803–0.902 |

*Note. For ischemic cholangiopathy versus DBD, the Mantel-Haenszel 95% CI is 0.16–3.01 (as reported in the main text and Table 2); the inverse-variance (DerSimonian-Laird) interval shown (0.386–3.766) is unstable owing to multiple double-zero studies, which is why the Mantel-Haenszel estimate is preferred.*

**Supplementary Table S2. Per-study raw data and reported hazard ratios**

*Events/total for the NRP arm and the comparator arm, by outcome and comparison, for every study in the dataset. “Excl.” marks rows excluded from the corresponding pooled analysis (retained descriptively). HR (95% CI) is shown where a study reported a time-to-event estimate. Technique-comparison rows (A- vs TA-NRP) are listed for completeness and were not pooled.*

| **Study** | **Comparison** | **Outcome** | **NRP e/N** | **Comparator e/N** | **HR (95% CI)** | **Excl.** |
| --- | --- | --- | --- | --- | --- | --- |
| Bababekov 2025 | vs non-NRP cDCD | Ischemic cholangiopathy | 1/86 | 7/74 | — |  |
| Bababekov 2025 | vs non-NRP cDCD | Primary non-function | 2/97 | 0/79 | — |  |
| Bababekov 2025 | vs non-NRP cDCD | Recipient death | 2/97 | –/79 | — |  |
| Bababekov 2025 | vs non-NRP cDCD | Graft loss | 3/97 | 3/79 | — |  |
| Bababekov 2025 | vs non-NRP cDCD | Early allograft dysfunction | 44/97 | 39/79 | — |  |
| Bababekov 2025 | vs non-NRP cDCD | Hepatic artery thrombosis | 0/97 | 3/79 | — |  |
| Bababekov 2025 | vs non-NRP cDCD | Other biliary complications | 5/97 | 7/79 | — |  |
| Bekki 2023 | vs non-NRP cDCD | Ischemic cholangiopathy | –/24 | –/1267 | — | Yes |
| Bekki 2023 | vs non-NRP cDCD | Primary non-function | –/24 | –/1267 | — | Yes |
| Bekki 2023 | vs non-NRP cDCD | Recipient death | –/24 | –/1267 | — | Yes |
| Bekki 2023 | vs non-NRP cDCD | Graft loss | 2/24 | –/1267 | — | Yes |
| Bekki 2023 | vs non-NRP cDCD | Early allograft dysfunction | –/24 | –/1267 | — | Yes |
| Bekki 2023 | vs non-NRP cDCD | Hepatic artery thrombosis | –/24 | –/1267 | — | Yes |
| Bekki 2023 | vs non-NRP cDCD | Other biliary complications | –/24 | –/1267 | — | Yes |
| Bluhme 2024 | vs DBD | Ischemic cholangiopathy | 0/18 | 2/28 | — |  |
| Bluhme 2024 | vs DBD | Primary non-function | 0/18 | 1/28 | — |  |
| Bluhme 2024 | vs DBD | Recipient death | 1/18 | 0/28 | — |  |
| Bluhme 2024 | vs DBD | Graft loss | 1/18 | 1/28 | — |  |
| Bluhme 2024 | vs DBD | Early allograft dysfunction | –/18 | –/28 | — |  |
| Bluhme 2024 | vs DBD | Other biliary complications | 1/18 | 3/28 | — |  |
| Brubaker 2024 | vs non-NRP cDCD | Ischemic cholangiopathy | 0/104 | 12/133 | — |  |
| Brubaker 2024 | vs non-NRP cDCD | Primary non-function | 0/106 | 2/136 | — |  |
| Brubaker 2024 | vs non-NRP cDCD | Recipient death | 1/106 | 3/136 | — |  |
| Brubaker 2024 | vs non-NRP cDCD | Graft loss | 1/106 | 7/136 | — |  |
| Brubaker 2024 | vs non-NRP cDCD | Early allograft dysfunction | 28/77 | 69/123 | — |  |
| Brubaker 2024 | vs non-NRP cDCD | Other biliary complications | 7/105 | 30/134 | — |  |
| Campo-Cañaveral 2023 | vs DBD | Ischemic cholangiopathy | 1/145 | 7/1162 | — |  |
| Campo-Cañaveral 2023 | vs DBD | Primary non-function | 6/145 | 24/1162 | — |  |
| Campo-Cañaveral 2023 | vs DBD | Recipient death | –/145 | –/1162 | — |  |
| Campo-Cañaveral 2023 | vs DBD | Graft loss | –/145 | –/1162 | — |  |
| Campo-Cañaveral 2023 | vs DBD | Early allograft dysfunction | –/145 | –/1162 | — |  |
| Campo-Cañaveral 2023 | vs DBD | Hepatic artery thrombosis | 5/145 | 52/1162 | — |  |
| Campo-Cañaveral 2023 | vs DBD | Other biliary complications | 12/145 | 98/1162 | — |  |
| Croome 2025 | vs non-NRP cDCD | Ischemic cholangiopathy | 0/62 | 50/297 | — |  |
| Croome 2025 | vs non-NRP cDCD | Primary non-function | 0/62 | 6/297 | — |  |
| Croome 2025 | vs non-NRP cDCD | Recipient death | –/62 | –/297 | — |  |
| Croome 2025 | vs non-NRP cDCD | Graft loss | –/62 | –/297 | — |  |
| Croome 2025 | vs non-NRP cDCD | Early allograft dysfunction | 7/62 | 187/290 | — |  |
| Croome 2025 | vs non-NRP cDCD | Hepatic artery thrombosis | 1/62 | 9/297 | — |  |
| Croome 2025 | vs non-NRP cDCD | Other biliary complications | –/62 | –/297 | — |  |
| Fernández-de la Varga 2022 | vs DBD | Ischemic cholangiopathy | 0/22 | 0/51 | — |  |
| Fernández-de la Varga 2022 | vs DBD | Primary non-function | 1/22 | 0/51 | — |  |
| Fernández-de la Varga 2022 | vs DBD | Recipient death | –/22 | –/51 | — |  |
| Fernández-de la Varga 2022 | vs DBD | Graft loss | –/22 | –/51 | — |  |
| Fernández-de la Varga 2022 | vs DBD | Early allograft dysfunction | –/22 | –/51 | — |  |
| Fernández-de la Varga 2022 | vs DBD | Hepatic artery thrombosis | 0/22 | 1/51 | — |  |
| Fernández-de la Varga 2022 | vs DBD | Other biliary complications | 0/22 | 1/51 | — |  |
| Gaurav 2022 | vs non-NRP cDCD | Ischemic cholangiopathy | 4/66 | 34/151 | — |  |
| Gaurav 2022 | vs non-NRP cDCD | Primary non-function | 0/69 | 6/164 | — |  |
| Gaurav 2022 | vs non-NRP cDCD | Recipient death | 2/69 | 8/164 | — |  |
| Gaurav 2022 | vs non-NRP cDCD | Graft loss | 2/69 | 15/164 | — |  |
| Gaurav 2022 | vs non-NRP cDCD | Early allograft dysfunction | 10/69 | 26/153 | — |  |
| Gaurav 2022 | vs non-NRP cDCD | Hepatic artery thrombosis | 1/69 | 12/156 | — |  |
| Gaurav 2022 | vs non-NRP cDCD | Other biliary complications | 11/69 | 27/153 | — |  |
| Hessheimer 2022 | vs non-NRP cDCD | Ischemic cholangiopathy | 6/545 | 24/258 | — |  |
| Hessheimer 2022 | vs non-NRP cDCD | Primary non-function | 16/545 | 15/258 | — |  |
| Hessheimer 2022 | vs non-NRP cDCD | Recipient death | 65/545 | 66/258 | 0.54 (0.373–0.781) |  |
| Hessheimer 2022 | vs non-NRP cDCD | Graft loss | 77/545 | 88/258 | 0.371 (0.267–0.516) |  |
| Hessheimer 2022 | vs non-NRP cDCD | Early allograft dysfunction | 81/545 | 60/258 | — |  |
| Hessheimer 2022 | vs non-NRP cDCD | Hepatic artery thrombosis | 22/545 | 19/258 | — |  |
| Hessheimer 2022 | vs non-NRP cDCD | Other biliary complications | 57/545 | 51/258 | — |  |
| Miñambres 2020 | vs DBD | Ischemic cholangiopathy | 0/16 | –/29 | — |  |
| Miñambres 2020 | vs DBD | Primary non-function | 2/16 | 0/29 | — |  |
| Miñambres 2020 | vs DBD | Recipient death | 2/16 | –/29 | — |  |
| Miñambres 2020 | vs DBD | Graft loss | –/16 | –/29 | — |  |
| Miñambres 2020 | vs DBD | Early allograft dysfunction | 3/16 | 5/29 | — |  |
| Miñambres 2020 | vs DBD | Hepatic artery thrombosis | 1/16 | 1/29 | — |  |
| Miñambres 2020 | vs DBD | Other biliary complications | –/16 | –/29 | — |  |
| Mohkam 2022 | vs non-NRP cDCD | Ischemic cholangiopathy | 2/68 | 3/34 | — |  |
| Mohkam 2022 | vs non-NRP cDCD | Primary non-function | 1/68 | –/34 | — |  |
| Mohkam 2022 | vs non-NRP cDCD | Recipient death | 0/68 | 2/34 | — |  |
| Mohkam 2022 | vs non-NRP cDCD | Graft loss | 3/68 | 3/34 | 0.67 (0.2–2.23) |  |
| Mohkam 2022 | vs non-NRP cDCD | Early allograft dysfunction | 14/68 | 3/34 | — |  |
| Mohkam 2022 | vs non-NRP cDCD | Hepatic artery thrombosis | 2/68 | 1/34 | — |  |
| Mohkam 2022 | vs non-NRP cDCD | Other biliary complications | 7/68 | 13/34 | — |  |
| Puttappa 2025 | vs non-NRP cDCD | Ischemic cholangiopathy | –/101 | –/137 | — | Yes |
| Puttappa 2025 | vs non-NRP cDCD | Primary non-function | 0/101 | 3/137 | — | Yes |
| Puttappa 2025 | vs non-NRP cDCD | Recipient death | –/101 | –/137 | — | Yes |
| Puttappa 2025 | vs non-NRP cDCD | Graft loss | –/101 | –/137 | 2.4 (1.1–5.4) | Yes |
| Puttappa 2025 | vs non-NRP cDCD | Early allograft dysfunction | –/101 | –/137 | — | Yes |
| Puttappa 2025 | vs non-NRP cDCD | Other biliary complications | –/101 | –/137 | — | Yes |
| Rodríguez 2022 | vs DBD | Ischemic cholangiopathy | 0/39 | 1/78 | — |  |
| Rodríguez 2022 | vs DBD | Primary non-function | 0/39 | 3/78 | — |  |
| Rodríguez 2022 | vs DBD | Recipient death | 2/39 | 7/78 | — |  |
| Rodríguez 2022 | vs DBD | Graft loss | 0/39 | 8/78 | — |  |
| Rodríguez 2022 | vs DBD | Early allograft dysfunction | 13/39 | 15/78 | — |  |
| Rodríguez 2022 | vs DBD | Hepatic artery thrombosis | 0/39 | 6/78 | — |  |
| Rodríguez 2022 | vs DBD | Other biliary complications | –/39 | –/78 | — |  |
| Rodríguez-Sanjuán 2019 | vs DBD | Ischemic cholangiopathy | 0/11 | 0/51 | — |  |
| Rodríguez-Sanjuán 2019 | vs DBD | Primary non-function | 1/11 | 1/51 | — |  |
| Rodríguez-Sanjuán 2019 | vs DBD | Recipient death | 1/11 | 2/51 | — |  |
| Rodríguez-Sanjuán 2019 | vs DBD | Graft loss | –/11 | –/51 | — |  |
| Rodríguez-Sanjuán 2019 | vs DBD | Early allograft dysfunction | –/11 | –/51 | — |  |
| Rodríguez-Sanjuán 2019 | vs DBD | Hepatic artery thrombosis | 1/11 | 7/51 | — |  |
| Rodríguez-Sanjuán 2019 | vs DBD | Other biliary complications | 2/11 | 13/51 | — |  |
| Ruíz 2021 | vs DBD | Ischemic cholangiopathy | 0/100 | 0/200 | — |  |
| Ruíz 2021 | vs DBD | Primary non-function | 0/100 | 2/200 | — |  |
| Ruíz 2021 | vs DBD | Recipient death | 4/100 | 22/200 | — |  |
| Ruíz 2021 | vs DBD | Graft loss | 2/100 | 9/200 | — |  |
| Ruíz 2021 | vs DBD | Early allograft dysfunction | 19/100 | 42/200 | — |  |
| Ruíz 2021 | vs DBD | Hepatic artery thrombosis | 1/100 | 3/200 | — |  |
| Ruíz 2021 | vs DBD | Other biliary complications | 5/100 | 12/200 | — |  |
| Savier 2020 | vs DBD | Ischemic cholangiopathy | 1/50 | 1/100 | — |  |
| Savier 2020 | vs DBD | Primary non-function | –/50 | –/100 | — |  |
| Savier 2020 | vs DBD | Recipient death | 1/50 | 1/100 | — |  |
| Savier 2020 | vs DBD | Graft loss | 1/50 | 5/100 | — |  |
| Savier 2020 | vs DBD | Early allograft dysfunction | 9/50 | 32/100 | — |  |
| Savier 2020 | vs DBD | Hepatic artery thrombosis | 0/50 | 5/100 | — |  |
| Savier 2020 | vs DBD | Other biliary complications | 7/50 | 16/100 | — |  |
| Schurink 2022 | vs DBD | Ischemic cholangiopathy | –/20 | –/81 | — |  |
| Schurink 2022 | vs DBD | Primary non-function | 0/20 | 1/81 | — |  |
| Schurink 2022 | vs DBD | Recipient death | –/20 | –/81 | — |  |
| Schurink 2022 | vs DBD | Graft loss | –/20 | –/81 | — |  |
| Schurink 2022 | vs DBD | Early allograft dysfunction | 5/20 | 25/81 | — |  |
| Schurink 2022 | vs DBD | Hepatic artery thrombosis | 0/20 | 4/81 | — |  |
| Schurink/De Goeij 2022 | vs non-NRP cDCD | Ischemic cholangiopathy | –/20 | –/49 | — |  |
| Schurink/De Goeij 2022 | vs non-NRP cDCD | Primary non-function | 0/20 | 1/49 | — |  |
| Schurink/De Goeij 2022 | vs non-NRP cDCD | Graft loss | –/20 | –/49 | — |  |
| Schurink/De Goeij 2022 | vs non-NRP cDCD | Early allograft dysfunction | 5/20 | 22/49 | — |  |
| Schurink/De Goeij 2022 | vs non-NRP cDCD | Hepatic artery thrombosis | 0/20 | 1/49 | — |  |
| Secanella 2023 | Technique (A- vs TA-NRP) | Ischemic cholangiopathy | 0/6 | 1/12 | — | Yes |
| Secanella 2023 | Technique (A- vs TA-NRP) | Primary non-function | 0/6 | 0/12 | — | Yes |
| Secanella 2023 | Technique (A- vs TA-NRP) | Recipient death | 0/6 | 1/12 | — | Yes |
| Secanella 2023 | Technique (A- vs TA-NRP) | Graft loss | 0/6 | 2/12 | — | Yes |
| Secanella 2023 | Technique (A- vs TA-NRP) | Early allograft dysfunction | 0/6 | 0/12 | — | Yes |
| Secanella 2023 | Technique (A- vs TA-NRP) | Other biliary complications | 3/6 | 2/12 | — | Yes |
| Viguera 2021 | vs DBD | Ischemic cholangiopathy | –/144 | –/447 | — |  |
| Viguera 2021 | vs DBD | Primary non-function | –/144 | –/447 | — |  |
| Viguera 2021 | vs DBD | Recipient death | 3/144 | 26/447 | 0.41 (0.14–1.21) |  |
| Viguera 2021 | vs DBD | Graft loss | –/144 | –/447 | — |  |
| Viguera 2021 | vs DBD | Early allograft dysfunction | –/– | –/– | — |  |
| Wisel 2023 | vs non-NRP cDCD | Ischemic cholangiopathy | –/133 | –/1219 | — | Yes |
| Wisel 2023 | vs non-NRP cDCD | Primary non-function | –/133 | –/1219 | — | Yes |
| Wisel 2023 | vs non-NRP cDCD | Recipient death | –/133 | –/1219 | — | Yes |
| Wisel 2023 | vs non-NRP cDCD | Graft loss | –/133 | –/1219 | — | Yes |
| Wisel 2023 | vs non-NRP cDCD | Early allograft dysfunction | –/133 | –/1219 | — | Yes |

**Supplementary Table S3. Sensitivity analysis excluding ex situ machine perfusion (NMP/HOPE).**

Comparison of the main pooled risk ratios (all pooled studies) with the analysis restricted to grafts free of ex situ perfusion, for NRP versus non-NRP cDCD. Every effect persists in the same direction and remains statistically significant. Reproduced in R/metafor 5.0-1 and independent Python (identical).

| **Outcome** | **Main analysis — RR (95% CI); k** | **Ex situ perfusion–free — RR (95% CI); k** | **I² (free)** |
| --- | --- | --- | --- |
| Ischemic cholangiopathy | 0.16 (0.09–0.28); 6 | 0.14 (0.08–0.26); 5 | 0% |
| Primary non-function | 0.51 (0.28–0.94); 6 | 0.49 (0.27–0.92); 5 | 0% |
| Recipient death | 0.46 (0.34–0.62); 4 | 0.47 (0.35–0.64); 3 | 0% |
| Graft loss | 0.42 (0.32–0.54); 5 | 0.41 (0.32–0.53); 4 | 0% |
| Hepatic artery thrombosis | 0.51 (0.30–0.85); 6 | 0.48 (0.28–0.83); 4 | 0% |
| Other biliary complications | 0.49 (0.32–0.73); 5 | 0.54 (0.37–0.79); 4 | 30% |
| Early allograft dysfunction | 0.65 (0.45–0.94); 7 | 0.58 (0.39–0.86); 5 | 78% |

*RR, risk ratio; k, number of contributing studies. Ex situ perfusion–free analysis excludes grafts managed with NMP or HOPE (studies 9, 11, 12). The NRP versus DBD comparison was inherently free of ex situ perfusion.*

**Supplementary Checklist 1. PRISMA 2020 checklist**

*Location refers to the section of the main manuscript or to the Supplementary Material (SM) where each item is reported.*

| **#** | **Checklist item** | **Reported in** |
| --- | --- | --- |
| **TITLE** | | |
| 1 | Identifies the report as a systematic review. | Title |
| **ABSTRACT** | | |
| 2 | Structured summary (single-paragraph, per journal style). | Abstract |
| **INTRODUCTION** | | |
| 3 | Rationale for the review in the context of existing knowledge. | Introduction |
| 4 | Explicit statement of objectives / questions (PICO). | Introduction; Methods |
| **METHODS** | | |
| 5 | Eligibility criteria and grouping for synthesis. | Methods; SM Methods 2 |
| 6 | Information sources and last search date. | Methods; SM Methods 1 |
| 7 | Full search strategy for all databases. | SM Methods 1 |
| 8 | Selection process (duplicate screening, arbitration). | Methods; SM Methods 1 |
| 9 | Data collection process (duplicate extraction). | Methods; SM Methods 4 |
| 10 | Data items (outcomes and definitions; other variables). | Methods |
| 11 | Risk-of-bias assessment (ROBINS-I; independent reviewers). | Methods; Results; Figures 2/S1 |
| 12 | Effect measures (RR; pooled incidences). | Methods; SM Methods 3 |
| 13 | Synthesis methods (model, heterogeneity, subgroups, sensitivity). | Methods; SM Methods 3 |
| 14 | Reporting-bias assessment. | Methods; SM Methods 3; Figure S2 |
| 15 | Certainty assessment (GRADE). | Methods; Results; Table 2 |
| **RESULTS** | | |
| 16 | Study selection (flow with reasons for exclusion). | Results; Figure 1 |
| 17 | Study characteristics. | Results; Table 1 |
| 18 | Risk of bias within studies. | Results; Figures 2/S1 |
| 19 | Results of individual studies. | SM Table S2 |
| 20 | Results of syntheses (per outcome, with heterogeneity). | Results; Tables 2–3; SM Table S1; Figure 3 |
| 21 | Reporting biases. | Results; SM Methods 3; Figure S2 |
| 22 | Certainty of evidence. | Results; Table 2 |
| **DISCUSSION** | | |
| 23 | Interpretation, limitations, implications. | Discussion |
| **OTHER INFORMATION** | | |
| 24 | Registration and protocol (PROSPERO CRD420261404939). | Methods; Title page |
| 25 | Support / funding and role of funders. | Funding |
| 26 | Competing interests. | Conflict of Interest |
| 27 | Availability of data, code and materials. | Data Availability; SM Methods 4 |

**Supplementary Checklist 2. MOOSE checklist (meta-analysis of observational studies)**

*Meta-analysis Of Observational Studies in Epidemiology. Location refers to the main manuscript or Supplementary Material (SM).*

| **MOOSE item** | **Reported in** |
| --- | --- |
| **Reporting of background** | |
| Problem definition | Introduction |
| Hypothesis statement | Introduction |
| Description of study outcomes | Methods (PICO) |
| Type of exposure / intervention | Introduction; Methods |
| Type of study designs eligible | Methods |
| Study population | Methods (PICO) |
| **Reporting of search strategy** | |
| Qualifications of searchers | SM Methods 1; Author Contributions |
| Search strategy and databases | SM Methods 1 |
| Use of hand searching | SM Methods 1 |
| List of included/excluded studies | Table 1; SM Methods 2; Figure 1 |
| Method of addressing non-English papers | Methods (English-language limit) |
| Handling of unpublished data / abstracts | Figure 1 (conference abstracts excluded) |
| Description of any contact with authors | Not required |
| **Reporting of methods** | |
| Rules of eligibility (rationale) | Methods; SM Methods 2 |
| Rating / appraisal of bias | Methods; SM Figure S1 (ROBINS-I) |
| Rationale for selecting/coding data | Methods; SM Methods 3/4 |
| Assessment of confounding | ROBINS-I; Discussion (limitations) |
| Assessment of study heterogeneity | Methods; SM Methods 3; SM Table S1 |
| Statistical methods in detail | Methods; SM Methods 3 |
| Provision for combining data | SM Methods 3 |
| **Reporting of results** | |
| Graphical summary | Figures 1–3; Figures S1–S2 |
| Table of descriptive information per study | Table 1; SM Table S2 |
| Sensitivity-analysis results | Results; SM Table S1 |
| Indication of statistical uncertainty | All estimates with 95% CI; PI in SM Table S1 |
| **Reporting of discussion** | |
| Quantitative assessment of bias | Discussion; ROBINS-I |
| Justification for exclusions | SM Methods 2; Figure 1 |
| Assessment of quality of included studies | GRADE (Table 2); ROBINS-I |
| **Reporting of conclusions** | |
| Consideration of alternative explanations | Discussion (EAD subgroup; confounding) |
| Generalization of conclusions | Discussion |
| Guidelines for future research | Discussion (Conclusion) |
| Disclosure of funding source | Funding |

**Capsule Sentence Summary**

In controlled DCD liver transplantation, normothermic regional perfusion markedly reduces ischemic cholangiopathy and improves graft and patient survival, bringing outcomes close to those of brain-death donors and supporting its role in safely expanding the donor pool.

**Graphical Abstract**

Prepared using the Transplant International template (16:9; Arial/Calibri ≥18 pt; ≥300 dpi). The editable file is provided separately (GraphicalAbstract_TI_FINAL.pptx).
